# Genomic and biogeographic characterisation of the novel prasinovirus *Mantoniella tinhauana* virus 1

**DOI:** 10.1111/1758-2229.70020

**Published:** 2024-10-11

**Authors:** Elvira Rey Redondo, Shara Ka Kiu Leung, Charmaine Cheuk Man Yung

**Affiliations:** ^1^ Department of Ocean Science The Hong Kong University of Science and Technology Hong Kong Hong Kong SAR

## Abstract

Mamiellophyceae are a ubiquitous class of unicellular green algae in the global ocean. Their ecological importance is highlighted in studies focused on the prominent genera *Micromonas*, *Ostreococcus*, and *Bathycoccus*. Mamiellophyceae are susceptible to prasinoviruses, double‐stranded DNA viruses belonging to the nucleocytoplasmic large DNA virus group. Our study represents the first isolation of a prasinovirus in the South China Sea and the only one to infect the globally distributed genus *Mantoniella*. We conducted a comparative analysis with previously identified viral relatives, encompassing morphological characteristics, host specificity, marker‐based phylogenetic placement, and whole‐genome sequence comparisons. Although it shares morphological and genetic similarities with established prasinoviruses, this novel virus showed distinct genetic traits, confining its infection to the species *Mantoniella tinhauana*. We also explored the global biogeography of this prasinovirus and its host by mapping metagenomic data and analysing their relationship with various environmental parameters. Our results demonstrate a pronounced link between the virus and its host, both found predominantly in higher latitudes in the surface ocean. By gaining an increased understanding of the relationships between viruses, hosts, and environments, researchers can better make predictions and potentially implement measures to mitigate the consequences of climate change on oceanic processes.

## Graphical abstract

Phylogenetic tree, virion morphology and global metagenomic abundance of *Mantoniella tinhauana* virus 1, a newly isolated and characterised prasinovirus.

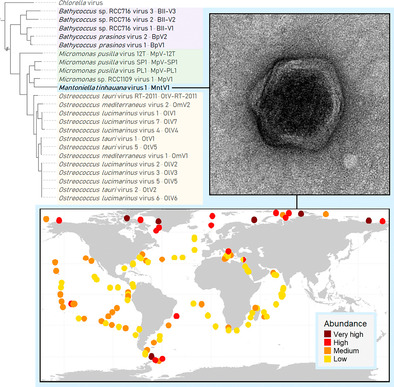

## INTRODUCTION

Prasinoviruses, belonging to the family Phycodnaviridae (recently re‐classified by Aylward et al., 2021 to the novel family Prasinoviridae, although this change has not yet been instated into the Current ICTV Taxonomy Release 2023), are lytic nucleocytoplasmic large DNA viruses (NCLDVs) that infect algae of the class Mamiellophyceae. To date, documented prasinovirus isolates are confined to three marine genera of the order Mamiellales (Bellec et al., 2009; Yung et al., 2022): *Micromonas*, *Ostreococcus*, and *Bathycoccus*. Viruses significantly influence biogeochemical cycles through two key mechanisms: the “viral shunt”, which recycles organic matter and maintains it within lower trophic levels, and the “viral shuttle”, which amplifies the efficiency of the biological pump, thereby increasing carbon sequestration at the ocean floor (Guidi et al., 2016; Lønborg et al., 2013; Sullivan et al., 2017; Wilhelm & Suttle, 1999). Prasinoviruses represent a significant portion of the marine eukaryotic virome (Hingamp et al., 2013), and their presence correlates with carbon export efficiency in the global ocean, with their impact varying positively or negatively based on host interactions and ecosystem dynamics (Kaneko et al., 2021).

Prasinoviruses, while being smaller entities within the NCLDV group, possess genomes ranging from 184 to 198 kb (Weynberg et al., 2017), which are still considerably large compared to their algal hosts (Grimsley et al., 2015). Remarkably, only a small number of prasinovirus particles, sometimes as few as nine, can span the diameter of certain Mamiellales algal cells, resulting in very low burst sizes, a distinctive feature of these infections (Grimsley et al., 2015). For instance, *Ostreococcus tauri*, which is among the smallest documented species within the Mamiellophyceae class, has a reported burst size of only 25 viruses per cell (Derelle et al., 2008). Despite their modest size, prasinoviruses emerge as one of the predominant groups of NCLDVs across the ocean (Ha et al., 2023; Hingamp et al., 2013).

A few complete prasinovirus genomes have been successfully sequenced and assembled, primarily focusing on *Ostreococcus* viruses (Derelle et al., 2008; Moreau et al., 2010; Weynberg et al., 2009, 2011), *Bathycoccus* viruses, and *Micromonas* viruses (Bachy et al., 2021; Finke et al., 2017; Moreau et al., 2010). While these genomes share a core set of genes, they exhibit greater genetic variability towards the terminal regions, which are characterised by variable repetitive regions (Moreau et al., 2010). Prasinovirus genomes, though generally less diverse than those of their algal hosts (Moreau et al., 2010; Weynberg et al., 2017), reflect a complex evolutionary history characterised by different rates of evolution, potential ancestral co‐speciation and subsequent viral species recombination and host‐virus gene transfers (Bellec et al., 2014; Grimsley et al., 2015; Moreau et al., 2010; Yau, Krasovec, et al., 2020). Despite this complexity, prasinoviruses maintain a high degree of host specificity, with some infecting different species, but always within the same host genus (Bellec et al., 2009; Clerissi et al., 2012). The occurrence of prasinovirus resistance or tolerance in cultured Mamiellales species is not uncommon, with spontaneous and stable resistance often developing (Thomas et al., 2011; Yau et al., 2016; Yau, Krasovec, et al., 2020).

All prasinoviruses possess a DNA polymerase B (*polB*) gene and multiple copies of major capsid protein (*mcp*) genes (Weynberg et al., 2017). These genes serve as key markers for various studies exploring the distribution of prasinoviruses at both local and global scales. Methods have included the extraction of marker sequences from metagenomic reads, the alignment of metagenomic reads to these markers, and the use of amplified marker genes (Bachy et al., 2021; Bellec et al., 2010; Chen et al., 1996; Chen & Suttle, 1995; Clerissi et al., 2012, 2014, 2015; Endo et al., 2020; Ha et al., 2023; Hingamp et al., 2013; Labbé et al., 2018; Maat et al., 2017; Manrique et al., 2012; Yau et al., 2015). The present study represents the first instance of mapping global metagenomic reads onto the complete genomes of both a prasinovirus and its algal host. This approach facilitates an in‐depth exploration of species‐specific biogeography on a global scale. By employing this genome‐wide mapping strategy, we circumvent the limitations and biases associated with gene amplification, database availability and the selection of particular genetic markers.

Our study reveals a novel prasinovirus infecting *Mantoniella tinhauana*, a globally distributed microalga with distinctive genomic characteristics. *M. tinhauana* stands out among Mamiellales species due to its enlarged genome and cell size, higher proportion of repetitive elements, and gene block duplications and rearrangements. Notably, it lacks the low‐GC outlier chromosomes common in related species. Despite its widespread distribution, *M. tinhauana* maintains a low‐level prevalence across diverse marine ecosystems worldwide (Rey Redondo et al., 2024). This virus, named *Mantoniella tinhauana* virus 1 (MntV1), represents the first instance of a prasinovirus infecting a host outside the well‐documented *Bathycoccus*, *Ostreococcus*, or *Micromonas* genera. Our study focuses on exploring the phylogenetic relationships and genomic differences between MntV1 and previously characterised prasinoviruses. Additionally, we delineate the global distribution of MntV1 and its algal host using a metagenomics‐based approach.

## EXPERIMENTAL PROCEDURES

### Virus isolation

*Mantoniella tinhauana* RCC11003 monoculture (1:10 subcultured approximately every 3 weeks for about 2 years since its initial isolation in June 2020) was grown in L1 medium (Guillard & Hargraves, 1993) in SPL cell culture flasks in a PHCbi shaking incubator (100 rpm) at room temperature (21–23°C) under a 12‐h light/dark cycle (30 μmol photons m^−2^ s^−1^) until it reached exponential growth phase (2–4 days).

To isolate viruses infecting *M. tinhauana*, a total of 24 seawater samples (50 ml) were collected from monthly samplings from three different locations around Hong Kong and kept in the dark at 4°C until use. Virus isolation methods are based on Bachy et al. (2021) without the concentration steps. Seawater samples were filtered through sterile Minisart High Flow 0.45 μm syringe filters (Sartorius) to remove any microbes, followed by addition (1 ml) to the exponentially growing *M. tinhauana* cultures (10 ml) in triplicate. Artificial seawater (24.55 g/L NaCl, 0.75 g/L KCl, 0.21 g/L NaHCO_3_, 1.904 g/L MgCl_2_, 1.11 g/L CaCl_2_, and 6.04 g/L MgSO_4_∙7H_2_O, based on Tabei et al., 2011, 0.22 μm filtered) was used as a control in triplicate alongside the experimental samples.

The cultures were incubated up to 2 weeks, during which their appearance was regularly inspected. Any sample showing distinct signs of lysis, indicated by a colour change from green to transparent (see Supplementary Figure S1 for photographic examples), was considered infected. One seawater sample caused lysis in *M. tinhauana* after a week and the culture exhibiting lysis was retained. The lysate was collected and filtered through a 0.45 μm syringe filter to exclude algal cells and kept at 4°C in the dark.

Viral particles in the lysate (1 week post‐infection) were stained with 0.5X SYBR Green I, incubated for 15 minutes in darkness at room temperature, and then quantified using flow cytometry on a FACSAria III machine (BD Biosciences). Quantification was based on forward scatter and SYBR Green fluorescence. Uninfected *M. tinhauana* exponentially growing cells (2 days post‐subculture) were also quantified by forward scatter and chlorophyll fluorescence. The concentration of cell culture in the exponential phase used throughout the methods was determined to be 2.94 million cells ml^−1^, whereas the concentration of virus was measured at 7.57 million cells ml^−1^. A 1:10 virus:host culture volume ratio was used, resulting in a multiplicity of infection of approximately 0.25. The viral lysate was serially diluted in artificial seawater and added in triplicate to exponentially growing *M. tinhauana* cultures in a 24‐well cell culture plate to determine the lowest concentration required for infection (visible lysis after a week). This lowest concentration was used in a plaque assay to isolate individual viral lysis plaques, based on published methods (Derelle et al., 2008, 2015). In brief, 5 ml of diluted viral lysate, 4 ml of exponentially growing *M. tinhauana* culture, 3.5 ml L1 medium and 1.5 ml of 1.5% liquid agarose gel were mixed and poured into triplicate 60 × 15 mm culture plates and incubated at room temperature for a week (again with artificial seawater as controls), keeping the air around the plate moist with distilled, autoclaved water‐dipped cotton balls. Individual plaques (transparent spots atop the pale green translucent agar) were picked and inoculated in exponentially growing liquid culture again, sequentially filtering the lysate through 0.45 μm and reinfecting larger volumes of host culture (1:10 virus:culture volume), up to 100 ml viral lysate. This lysate was filtered, kept at 4°C in the dark and used for the characterisation steps below.

### Transmission electron microscopy

Transmission electron microscopy (TEM) methods employed in this study were based on published references (Bachy et al., 2021; Monninger et al., 2016). Fresh viral lysate was initially filtered through 0.45 μm filters to separate the viral fraction. The filtered lysate was then concentrated 15 times using Amicon 100 kDa MWCO Ultra Centrifugal Filter units (Merck). It is important to note that *Mantoniella tinhauana* surface scales, which cover both the cell body and flagella, frequently detach during sample preparation. This phenomenon is not unique to infected samples; it is also observed in uninfected, unfiltered *M. tinhauana* cultures (Rey Redondo et al., 2024). These scales, approximately 200 nm in diameter, can pass through the 0.45 μm filter used in the concentration process. Consequently, they consistently appear alongside virus particles in TEM images. Subsequently, 8 μl of concentrated lysate was applied onto carbon‐coated 400 mesh copper TEM grids (EM Resolutions), and allowed to dry for 15 min at room temperature. Excess volume was carefully removed using blotting paper. This process was repeated two more times, followed by two rounds of washing with 8 μl distilled water. For negative staining, the grids were then treated with 10 μl 2% uranyl acetate for 15 s, followed by another round of drying. Finally, imaging was carried out on a JEOL JEM‐2010F Transmission Electron Microscope at 200 kV acceleration.

### Host range

To assess cross‐infectivity, 15 species of Mamiellophyceae (*Mantoniella tinhauana* RCC11003, *Mantoniella squamata* RCC395 and RCC417, *Mantoniella* sp. RCC10708, *Mantoniella beaufortii* RCC2288 and RCC2497, *Mamiella gilva* RCC6674, *Micromonas commoda* RCC447, *Micromonas bravo* RCC418, *Micromonas pusilla* RCC834, *Ostreococcus lucimarinus* RCC3401, *Ostreococcus tauri* RCC4221, *Ostreococcus mediterraneus* RCC6889, *Bathycoccus prasinos* RCC2486, and *Bathycoccus calidus* RCC715) were cultured (Vaulot et al., 2004). Cultures of each species were prepared in transparent 96‐well microplates (Thermo Fisher Scientific), and fresh filtered viral lysate (MntV1) or artificial seawater (control) was added in triplicate to the exponentially growing algal cultures. The microplates were then incubated for 2 weeks under the optimal growth conditions for each species. To monitor the level of algal cell lysis, optical density at 750 nm was measured periodically as in Bachy et al. (2021) and Hotos (2023) (on days 1, 2, 3, 5, 7, 9, 12, and 14) using a CLARIOstar Plus microplate reader (BMG Labtech). These measurements allowed for the quantification of algal cell lysis caused by MntV1 infection (e.g., see Supplementary Figure S1B).

### *polB* amplification and phylogeny

To confirm the presence of the algal virus and perform preliminary taxonomic classification, PCR was conducted on the viral lysate. A fragment of the viral *polB* gene was amplified using the VpolAS4 (5′‐GAR GGI GCI ACI GTI YTN GA‐3′)–VpolAAS1 (5′‐CCI GTR AAI CCR TAI ACI SWR TTC AT‐3′) primer set as in references (Bachy et al., 2021; Clerissi et al., 2014). The PCR started with an initial 3 min at 94°C, then 35 rounds of 94°C for 30 s, 50°C for 30 s and 72°C for 1 min, followed by final extension at 72°C for 5 min. The amplified DNA was purified using a QIAquick PCR Purification Kit (Qiagen) and sent for Sanger sequencing with TechDragon (Hong Kong). The obtained *PolB* sequence fragment (300 bp) was blastn searched against the NCBI‐nt database using Geneious Prime v2022.2.2, yielding high‐level matches to many prasinovirus *PolB* genes.

For taxonomic classification, a total of 23 *polB* sequences were aligned, including MntV1‐*polB* and 22 prasinovirus representative *polB* sequences extracted from NCBI genomes (as listed in Supplementary Table TS2, all saltwater prasinovirus sequences available from NCBI, based on Bachy et al., 2021) as well as one outgroup (*Paramecium bursaria Chlorella* virus 1‐*polB*) using MAFFT v7.453 (Katoh & Standley, 2013). Low‐quality positions with gaps in over 50% of the sequences were removed using Goalign clean sites v0.3.5 (Lemoine & Gascuel, 2021). A maximum likelihood (ML) tree was built using the web version of IQ‐TREE (Trifinopoulos et al., 2016), simultaneously generating Shimodaira‐Hasegawa‐like approximate likelihood ratio test branch support values from 1000 replicates and performing Markov chain Monte Carlo iterations for 1,000,000 generations sampling every 100 generations with 100,000 burn‐in length, as in Yau, dos Santos, et al. (2020). The resulting tree was rooted and visualised on Interactive Tree Of Life (iTOL) v5 (Letunic & Bork, 2021).

### Whole genome DNA extraction, sequencing, assembly and annotation

Viral DNA was extracted from the single‐colony 100 ml lysates using a modified CTAB protocol based on references (Clerissi et al., 2014; Winnepenninckx et al., 1993). Subsequently, the extracted viral DNA was sequenced on the Novaseq 6000 PE150 platform by Novogene (Hong Kong) using Illumina sequencing technology. After sequencing, the obtained reads were subjected to trimming using trimmomatic v0.39 (Bolger et al., 2014). Quality control was performed using fastp v0.23.2 (Chen, Zhang, et al., 2018; Chen, Zhou, et al., 2018). The processed reads were then de novo assembled by SPAdes v3.13.1 (Prjibelski et al., 2020) to generate draft genomes. To ensure the quality and integrity of the draft genomes, extraneous sequences were removed using Anvi'o v7.1 (Eren et al., 2015), and short Illumina reads were mapped back onto viral contigs using BBMap v38.96 (Bushnell, 2016) with a very high identity threshold (minid = 0.99). The mapped output reads were reassembled with SPAdes and decontaminated once again with Anvi'o.

To confirm that the three viral genomes obtained from three individual lysis plaques cultured belonged to the same species of prasinovirus (Figueras et al., 2014), the average nucleotide identity (ANI) was calculated using skani v0.1.0 (Shaw & Yu, 2023). The ANI for the three pairs of genomes was 99.5–100%, establishing them as a single strain.

tRNA genes in the novel genome were identified with tRNAscan‐SE On‐line (Lowe & Chan, 2016). Terminal inverted repeats (TIRs) were searched for in the novel genome using the Repeat Finder plugin v1.0.1 on GeneiousPrime. Short reads were mapped onto the genome split into 10,000 bp sections using BBMap at 95% identity and compiled using pileup.sh from the BBMap suite to test for potential terminal misassembly of repeats.

In addition to the newly assembled MntV1 genome, 22 prasinovirus genomes (listed in Supplementary Table TS2) and five outgroup chlorovirus genomes (the closest relatives of prasinoviruses) as in Bachy et al. (2021) were downloaded from NCBI. The 28 genomes (including the novel MntV1) were annotated using prokka v1.13 (Seemann, 2014) with the “–kingdom Viruses” tag.

### NCLDV phylogenomics

The proteomes generated by prokka were subjected to NCLDV‐markersearch (Moniruzzaman et al., 2020) to identify key NCLDV marker genes, confirming the completeness of the MntV1 assembly and to perform a phylogenetic comparison to the other species (Ha et al., 2023). All NCLDV marker sequences identified were concatenated for each species, aligned using MAFFT, and low‐quality positions with gaps were removed using Goalign clean sites. Using the cleaned alignment, an ML tree was built with IQ‐TREE and visualised on iTOL as described previously.

### Proteome characterisation and comparison

To compare the protein composition of various species, a comparative analysis was performed using Orthofinder v2.5.4 (Emms & Kelly, 2019) with the 23 prasinovirus proteomes. The orthogroups, along with their corresponding gene numbers, were visualised using R packages ggplot2 (Wickham, 2006) and reshape2 (Wickham, 2007) to create a heatmap representation. CAFE5 v1.1 (Mendes et al., 2021) was used to estimate gene family evolution (expansions and contractions) among the orthogroups of a subset of 10 species of prasinovirus, generating an ultrametric tree. Pie charts of the expansions and contractions for each species were added using meta‐chart.com.

The whole MntV1 proteome as well as the orthogroups found to be unique, expanded or contracted in MntV1 were functionally annotated using HMMER v3.3 (Eddy, 2011), which generated Pfam domain annotations (Mistry et al., 2021). These were used to identify and quantify specific protein groups. Some missing core genes which had been Pfam misannotated were matched to known gene sequences and annotated manually on Geneious Prime.

### Gene transfer

To identify probable gene transfers from the host to the novel virus, the MntV1 protein sequences were blastp searched against a custom database consisting of the *M. tinhauana* proteome sequence, deduplicating and keeping only hits with an E‐value below 10^−15^ and percentage identical positions above 50%. These thresholds were chosen as a compromise between those used in different studies (Bachy et al., 2021; Finke et al., 2017; Zhu et al., 2011), all of which use blast E‐values between 10^−5^ and 10^−20^ and identity between 25 and 50%. We wanted to obtain highly similar matches irrespective of length that were very significant. The MntV1 proteome was also blastp searched against the full NCBI‐nr database via DIAMOND v2.0.14.152 (Buchfink et al., 2021) and quality controlled as before, to identify gene transfer events from other sources.

Pie charts were created (on Excel) to depict the best‐blastp‐hit source organisms for all MntV1 protein‐coding genes as well as the subset of “semi‐conserved” genes. An ML tree was generated of the two MntV1‐unique FG‐GAP genes and their blastp best hits as run on Geneious Prime against the nr database, following the same alignment and visualisation methods as for the *polB* and NCLDV ML trees above.

### Global biogeography

Tara Oceans metagenomic data for surface (5 m depth) samples was downloaded from the ENA website in 2023, for the small DNA virus fraction (<0.22 μm: PRJEB4419‐global and PRJEB9742‐polar), the fraction for prokaryotes and large DNA viruses (0.22–3 μm: PRJEB1787‐global and PRJEB9740‐polar), and the protist size fraction (>0.8 μm: PRJEB4352‐global and PRJEB9691‐polar), together with their corresponding environmental data. These metagenomic reads were mapped onto the full MntV1 genome using BBMap at high identity (minid = 0.95) and compiled as above. For the study of virus–host co‐occurrence, metagenomic surface reads from the protist size fraction were also mapped against the *Mantoniella tinhauana* genome. In order to compare mapping of reads with different sequencing depths and mapping against differently sized (MntV1 and *M. tinhauana*) genomes, mapping values were calculated as Reads per Kilobase per Million (RPKM) following the equation:

$$\text{RPKM}=\text{mapped reads}÷\left(\;\frac{\text{genome length}}{1,000}\times \frac{\text{total reads}}{1,000,000}\;\right)$$

RPKM and coordinates were collated and the ggplot2 (Wickham, 2006) and scatterpie (Yu & Yu, 2018) R packages were used to plot four world maps. Median environmental values were obtained and Spearman's rank correlation coefficients (SRCCs) and corresponding *p*‐values calculated for every pair of variables in R. Highly collinear variables (SRCC over 0.5) were removed (conductivity, density, oxygen, and chlorophyll *a*). Temperature, salinity and nitrate levels (the latter had some data missing, so fewer samples were included) SRCCs with MntV1‐RPKM and host‐RPKM were retained and pairwise scatterplots plotted with ggpairs (GGally) (Schloerke et al., 2018).

## RESULTS AND DISCUSSION

### Isolation of a novel *Mantoniella tinhauana* ‐infecting prasinovirus

We collected 24 surface seawater samples at several sites around Hong Kong and tested them for the presence of lytic viruses infecting the recently isolated Mamiellophycean alga *Mantoniella tinhauana* (Rey Redondo et al., 2024). Among these, only a single sample, collected in April 2021 from Lau Fau Shan in the Pearl River Estuary to the northwest of Hong Kong (22°28′09.0″N 113°58′50.1″ E, temperature 24.3°C, 22.4 ppt salinity, and pH 8.3), caused clear infection and cell lysis in the cultures of *M. tinhauana*. This location coincides with the original discovery site of *M. tinhauana*, although the alga was found in a different season (June 2020) (Rey Redondo et al., 2024). Through isolation from this lytic event, we were able to identify and characterise a distinct viral strain, thereby confirming the presence of a novel prasinovirus species.

### Morphology of MntV1

**FIGURE 1:**
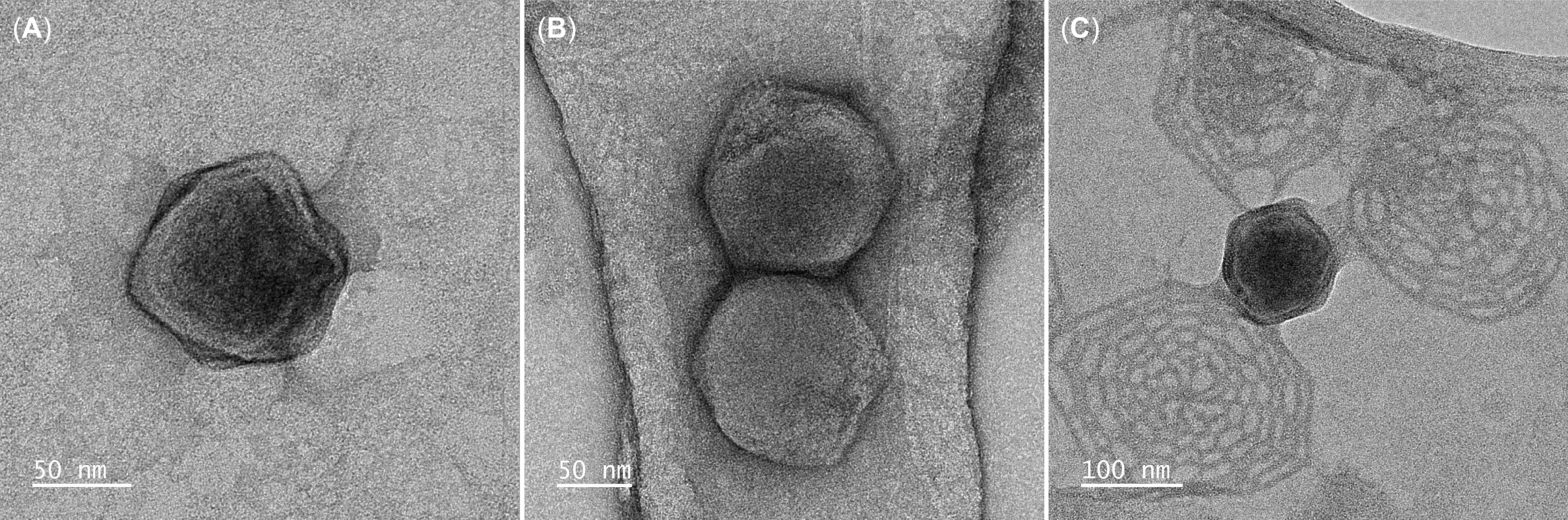
TEM images of MntV1 virions. (A) Isolated virion. (B) Aggregated virions. (C) Virions attached to loose host cell surface scales.

TEM imaging of MntV1 (Figure 1) revealed the characteristic icosahedral structure observed in all other prasinoviruses, consistent with the size range previously described (Bachy et al., 2021; Weynberg et al., 2017). The average diameter of MntV1 virions (*n* = 15) was 120.7 nm, with the smallest and largest virions at 105.5 and 142.6 nm, respectively, and a standard deviation of 11.7 nm. A notable observation from the TEM images is the aggregation of virions, as exemplified in Figure 1B. This aggregation phenomenon has previously been observed in TEM images of prasinoviruses (Derelle et al., 2015; Weynberg et al., 2017). However, it remains unclear whether this is merely an artefact of TEM preparation—potentially due to filtering, concentration, or using negative cationic stain (De Carlo & Harris, 2011)—or if it also occurs naturally in seawater, shaping infection dynamics (Pradhan et al., 2022). Further in vivo research is required to determine the significance of prasinoviral aggregation.

Figure 1C showcases a virion surrounded and adhered to by detached host surface scales. Scale formation in Mamiellophyceae is associated with the enzymatic activity of glycosyltransferases and sialidases (Moreau et al., 2012; Rey Redondo et al., 2024; van Baren et al., 2016), which mediate the addition and removal of sugar moieties on the cell surface, suggesting that these scales are glycan‐coated. Glycans are recognised as key elements in viral attachment and entry, serving as primary or secondary cell surface receptors, and are also implicated in the process of egress (Koehler et al., 2020). While the precise molecular mechanisms governing the entry of prasinovirus into their Mamiellophyceae hosts are not yet fully understood, and it remains uncertain whether viral capsids are glycosylated (Speciale et al., 2022), there is compelling evidence supporting the necessity of glycan‐lectin interactions during the entry process for these viruses (Weynberg et al., 2009, 2017). In scaled species of Mamiellales such as *Bathycoccus* and *Mantoniella*, where scales cover the entire cellular surface, and also the flagella in *Mantoniella* species (Bachy et al., 2021; Moreau et al., 2012; Yau, dos Santos, et al., 2020), it is plausible to infer that the initial virus–host contact might involve binding to these glycan‐rich scales. Thus, Figure 1C provides an illustration of what is likely a typical interaction at the virus–host interface within this group of organisms, although once more, it remains to be determined whether this virion‐scale binding occurs in vivo.

### MntV1 has high host specificity

Our host range experiments involving MntV1 and an array of 15 Mamiellophyceae species collected from diverse global sites (see Supplementary Table TS1) revealed a unique specificity: MntV1 demonstrated lysis capability solely with *Mantoniella tinhauana*. This specificity aligns with the pattern reported in prior studies, where prasinoviruses are often observed to infect multiple, but closely related host species within the same genus (Bellec et al., 2009; Clerissi et al., 2012). MntV1, however, exhibits a higher level of host specificity than is generally characteristic of prasinoviruses, with infection limited to a single species in our study. It is conceivable that MntV1 possesses the potential to infect other *Mantoniella* species or closely related strains that were not available for testing in our study, including those that have not yet been successfully cultured. This possibility underscores the necessity for ongoing exploration into the diversity of Mamiellophyceae and their associated viruses. The narrow host range of MntV1 suggests a highly evolved and specialised virus–host interaction, which could provide insights into the mechanisms of host specificity and virus evolution.

### Phylogenetic analysis of MntV1

In our investigation of the prasinovirus strain MntV1, we first focused on the DNA polymerase B gene (*polB*), a common genetic marker in prasinovirus research. A blastn search of the partial *polB* gene sequence from MntV1 revealed its highest similarity to the *Micromonas commoda* virus isolate McV‐KB1 (OQ440138), with a pairwise identity of 96.5%. We then constructed a phylogenetic tree using the *polB* gene sequences from a representative range of 23 prasinoviruses (Supplementary Table TS2) and one chlorovirus as an outgroup (Supplementary Figure S2), which positioned MntV1 within the *Micromonas* virus lineage and indicated a close relationship with the *Ostreococcus* virus lineage. However, this *polB*‐based phylogenetic tree yielded low branch support, reflecting the inherent limitations of using single‐gene markers to deduce phylogenetic relationships.

**FIGURE 2:**
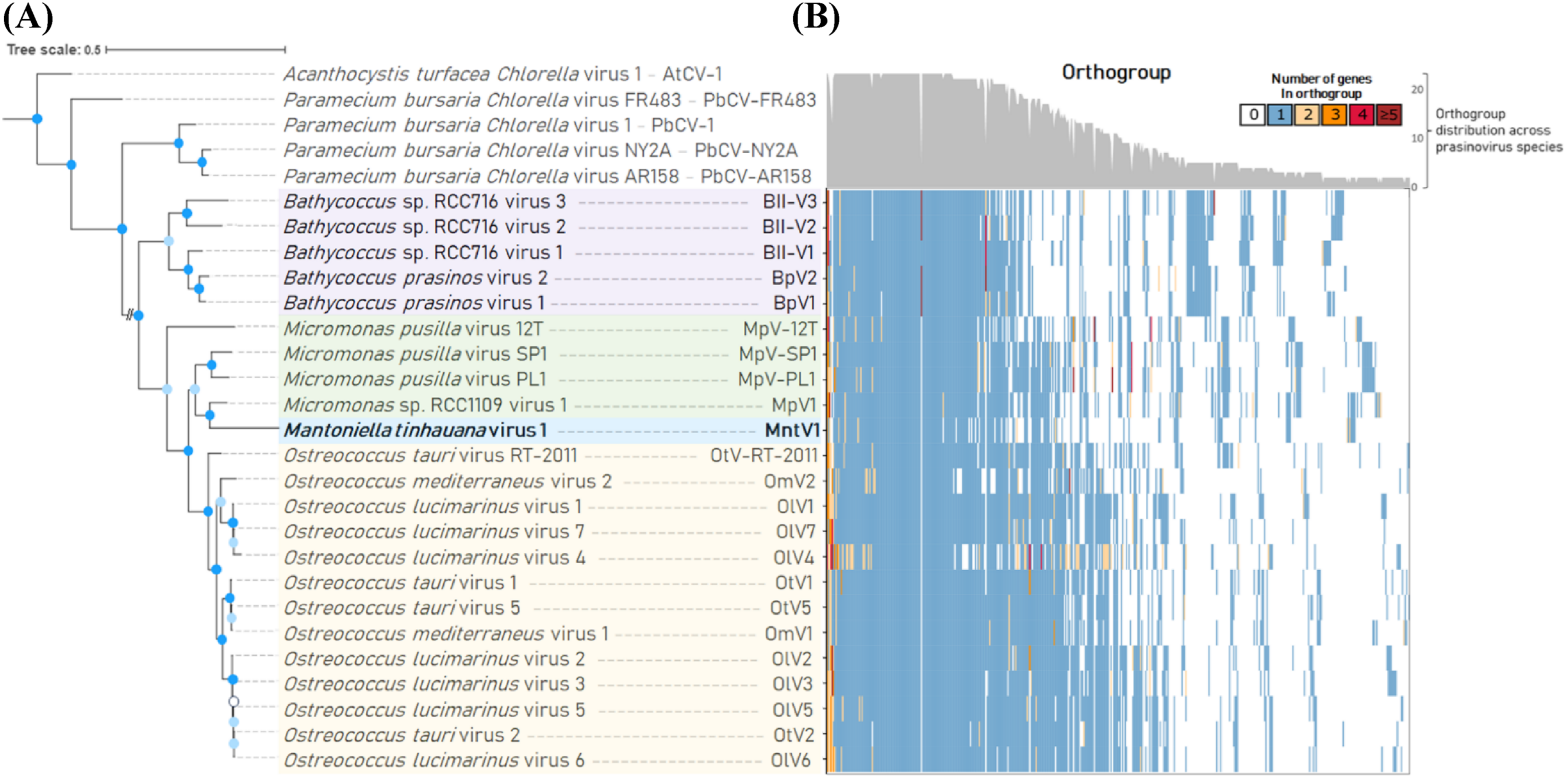
Concatenated NCLDV marker gene maximum likelihood tree and orthogroup distribution among prasinoviruses. (A) *Chlorella* viruses were used as an outgroup for tree construction and the branch connecting the prasinoviruses and the outgroup was truncated for display simplicity. Nodes with 100% support from both the Shimodaira‐Hasegawa (SH)‐like value and Bayesian posterior probability values are indicated with bright blue circles, whereas nodes with high but not full (>80%) SH‐like support and 100% Bayesian support are marked in light blue. Only one, marked in white, had lower support. Boxes of different colour encase the species corresponding to each host genus. The new *Mantoniella tinhauana* virus is highlighted in bold. (B) Orthogroups presence in all 23 prasinovirus species and orthogroup genes heatmap. The y‐axis is the prasinovirus species name, whereas the x‐axis shows each individual orthogroup. Number of genes in each orthogroup is shown in different colours. The bar chart at the top right represents the number of species, out of the 23, that contain each orthogroup, with the scale indicated on the right.

To obtain a more robust phylogeny, we systematically screened for NCLDV‐specific marker gene sequences (Moniruzzaman et al., 2020) within the genomes of the 23 prasinoviruses examined, along with five chlorovirus species as an outgroup. Our analysis revealed that seven out of the nine NCLDV markers were consistently found across the studied prasinovirus and chlorovirus genomes, including in MntV1: NCLDV major capsid protein, DEAD/SNF2‐like helicase, DNA polymerase B, Transcription initiation factor IIB, DNA topoisomerase II, Packaging ATPase, and Poxvirus Late Transcription Factor VLTF3 were present, while two DNA‐directed RNA polymerase subunits, alpha and beta, were not (Moniruzzaman et al., 2020). This is as expected, since prasinoviruses and chloroviruses, unlike other NCLDVs, do not carry their own DNA‐dependent RNA polymerase and must make use of the host cell's in the nucleus, as established previously (Moreau et al., 2010). Notable exceptions were observed in two prasinovirus species, OmV2 and BII‐V2, each lacking one of the seven conserved marker genes. Based on the concatenated sequences of the conserved NCLDV markers, we constructed a maximum likelihood (ML) phylogenetic tree, which provided robust branch support (Figure 2A), in contrast to the initial *polB*‐based phylogenetic inferences (Supplementary Figure S2). The ML tree confidently places MntV1 at a phylogenetic nexus between the *Micromonas* and *Ostreococcus* virus groups, establishing a clade that is closely related to the *Micromonas* virus clade. The NCLDV marker‐ML tree thus offers a more resolved view of MntV1's phylogenetic placement, enhancing our comprehension of its evolutionary relationships within the prasinovirus lineage.

### Genomic features of MntV1

MntV1 emerges as a newly identified prasinovirus with a relatively compact genome, ranking third in genome size among the 23 prasinoviruses analysed (see Supplementary Table TS2), at 177,820 bp. In line with its smaller genome, MntV1 also possesses fewer protein‐coding sequences, with 227 CDS (coding sequences), placing it third from last in gene count within the comparative prasinovirus dataset. Notably, the gene density of MntV1, expressed as the ratio of genome length to CDS, does not align with its position in terms of genome size or gene count. This discrepancy points to a potential unique genomic organisation in MntV1, differentiating it from other prasinoviruses. Furthermore, the genomic GC content of MntV1 falls within the typical range for prasinoviruses, which is generally lower than the GC content of their algal hosts (Moreau et al., 2010; Rey Redondo et al., 2024).

Members of the prasinoviruses have typically been found to contain terminal inverted repeats (TIRs) in the first and last 10,000 bp of the linear genome (Bachy et al., 2018; H. Chen, Zhang, et al., 2018; Derelle et al., 2018; Moreau et al., 2010). However, our analysis of the MntV1 genome's terminal regions revealed no TIRs. The short read sequencing data used in this study, featuring 150 bp reads—which are shorter than the smallest known prasinovirus TIRs of 250 bp in BpV2 (Moreau et al., 2010)—may lead to misassemblies including collapsed repeats (Treangen & Salzberg, 2012). Consequently, there is a possibility that the terminal regions of our de novo assembly might be incorrect. To investigate this, we mapped sequencing reads to the assembled genome divided into 10,000 bp sections at both ends, and analysed the read recruitment. The recruitment rates at these terminal sections did not exceed those in other parts of the genome, indicating a likely accurate assembly without misassembled repeats. This suggests that MntV1 lacks TIRs. Future studies utilising long‐read sequencing could confirm these findings.

### Unique orthogroups in MntV1

Hierarchical clustering was used to analyse the patterns of orthologous genes across the 23 prasinovirus genomes (listed in Supplementary Table TS2). The clustering results were consistent with the phylogenetic tree previously constructed. Within this dataset, MntV1 was distinguished by having the fewest orthogroups (198), in contrast to OlV2, which had the largest number of orthogroups with 260 (Figure 2B and Supplementary Table TS3). The lower number of orthogroups in MntV1 can be attributed to its smaller number of genes (227) and the relatively low proportion of these genes assigned to orthogroups (208 or 91.6%). Despite its lower gene count, MntV1 features four genes assigned to species‐specific orthogroups, making up 1.8% of its gene repertoire. Among all 23 prasinoviruses examined, only six species harboured species‐specific orthogroups, indicating that unique orthogroups are quite rare in prasinoviruses, with only eight unique orthogroups identified out of 452 in total. Approximately half of MntV1's orthogroups (93) were shared among all prasinoviruses examined, indicating a substantial conservation of core genetic functions.

To obtain deeper insights into the unique features of MntV1, we focused on the genes within the two orthogroups exclusive to this virus (Supplementary Table TS4A). We annotated these genes using the Pfam database to determine their potential functions by identifying their associated protein domains. Our analysis revealed that both MntV1‐specific orthogroups contain distinct FG‐GAP repeat domains. These domains, which are widespread among various proteins in both eukaryotes and bacteria, are crucial for numerous biological processes, key among them surface protein interactions, integrin activity, and host‐pathogen recognition (Al‐Shami et al., 2013; Loftus et al., 1994). For example, FG‐GAP domains have been identified as components of integrins on the surface of shrimp cells with which White Spot Syndrome Virus surface proteins interact (Sun et al., 2014). Similarly, in other multicellular hosts, viruses often bind to surface integrins, though these studies do not specifically reference the FG‐GAP domain (Triantafilou & Triantafilou, 2001). This pattern suggests a common mechanism where viral proteins attach to integrin receptors that contain FG‐GAP domains on host cells. To our knowledge, there is only one other virus, the amoeba‐infecting NCLDV medusavirus, with its own FG‐GAP repeat domain‐containing ORF (Yoshikawa et al., 2019), indicating a reversal of the typical interaction pattern. In this unusual case, the virus itself harbours the FG‐GAP domain, potentially binding to different host surface proteins, similar to what we observe in MntV1. The presence of FG‐GAP repeat domains in MntV1 hints at potential specialised mechanisms for interacting with its host compared to the other prasinoviruses. Identifying these domains is a pivotal stride towards unravelling the interaction modalities of MntV1 with its host organism and its ecological context. Further investigations into the specific functions and broader implications of the FG‐GAP repeat domains within MntV1 will not only enhance our understanding of the viral‐host interactions, but also provide valuable insights into the ecological dynamics and evolutionary strategies of prasinoviruses.

### Genomic divergences in MntV1

**FIGURE 3:**
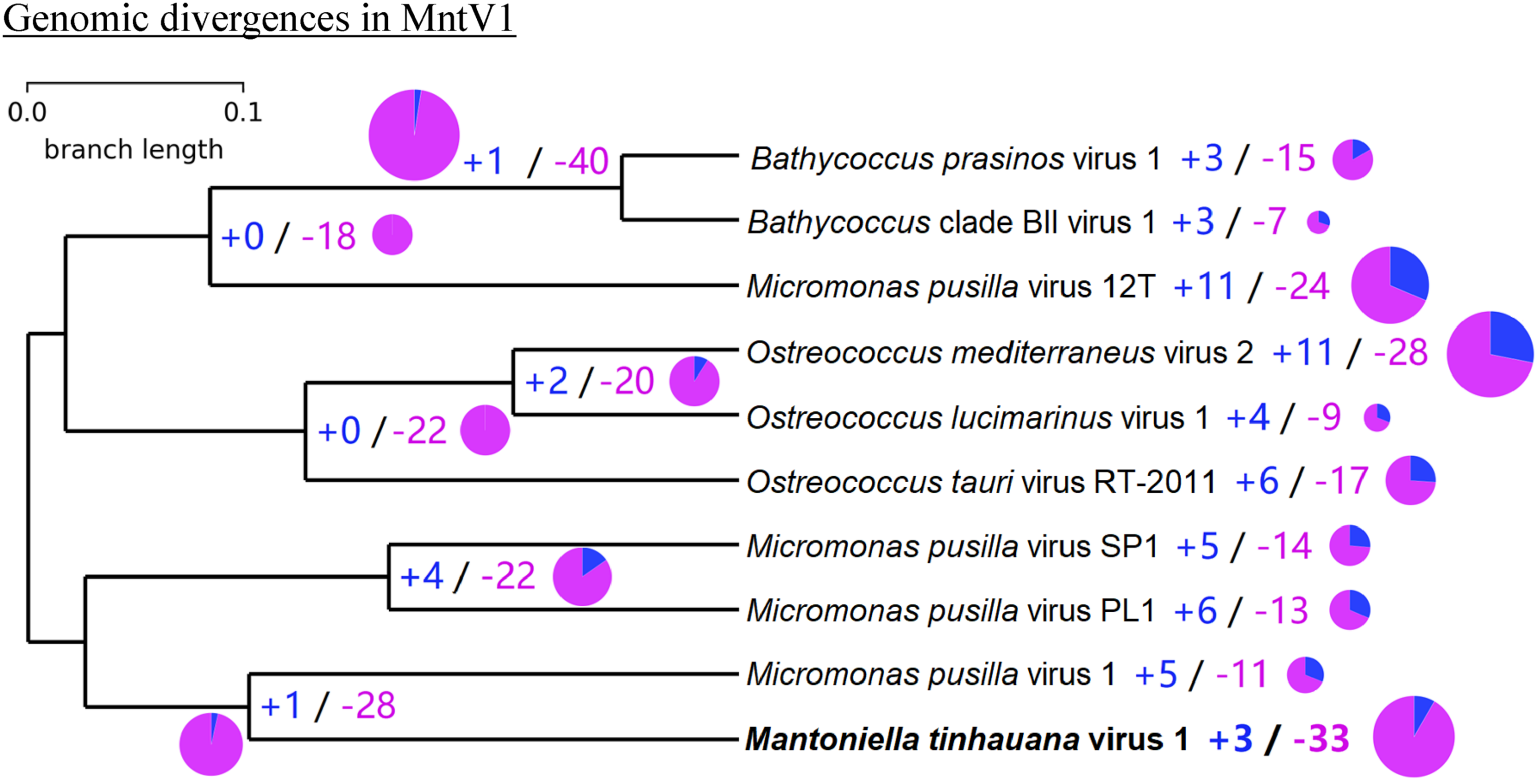
Ultrametric tree of the 10 prasinovirus species from the expansions (blue) and contractions (violet) analyses. MntV1 is highlighted in bold. The number of contractions and expansions for each branch is displayed in pie chart form, with the size of each chart corresponding to the combined total of contractions and expansions.

A genome‐wide analysis was performed to identify unique genes and orthogroups that were expanded or contracted in MntV1 relative to other prasinoviruses. Due to bioinformatic constraints, we focused on a subset of 10 prasinovirus genomes, representatives infecting various host species, and constructed the expansions and contractions tree seen in Figure 3. Interestingly, MntV1 exhibited more gene contractions than expansions. The analysis identified expansions in only three orthogroups within MntV1. Further examination of these orthogroups using Pfam annotation (Supplementary Table S2B) revealed that only one orthogroup showed an expansion (duplication) of a domain, specifically the NFACT protein RNA binding domain. This domain was identified in seven out of the 10 species evaluated, excluding the *Bathycoccus* viruses and MpV1.

The NFACT protein, which originates from eukaryotes, is involved in the ribosome‐associated quality control (RQC) pathway. This pathway is responsible for the degradation of stalled nascent polypeptides (Kostova et al., 2017; Shao et al., 2015). This suggests that some prasinoviruses may utilise this pathway to hijack the host's ribosomal machinery, potentially enhancing the translation of viral proteins and facilitating more efficient viral propagation. MntV1's expanded use of this domain may indicate a strategy for accelerated virion production compared to that in other prasinoviruses, although experimental validation is necessary to confirm this hypothesis. While manipulation of the RQC pathway by eukaryotic viruses has been documented (Lu, 2022), this study marks the first observation of such modifications in prasinoviruses.

The NFACT protein RNA binding domain shares high similarity with the domains found in fibronectin/fibrinogen binding protein. This similarity raises the possibility that prasinoviruses may instead possess a protein akin to fibronectin/fibrinogen binding proteins. A previous study identified NFACT as an accessory gene in the Coccolithovirus pangenome, although the study also highlighted the high similarity between NFACT and fibronectin binding domains and was unable to definitively determine the function of this protein in algal host infection (Lobb et al., 2023). Fibronectin binding proteins, found in vertebrate pathogens including both bacteria (Menzies, 2003) and viruses (Keski‐Oja et al., 1987), facilitate attachment and entry into host cells by binding to extracellular fibronectin and engaging with integrins (He et al., 2018; Proctor, 1987). Although this putative function has not been documented in algae but rather in multicellular eukaryotes, it could be related to the unique integrin‐related genes detected in MntV1. One hypothesis proposed that glycosyltransferase family 2 proteins in viruses infecting Mamiellales might trigger host cell aggregation within a hyaluronan network, mimicking extracellular matrix conditions observed in multicellular eukaryotes (Kaneko et al., 2021; Van Etten et al., 2017). This hypothesised function, however, based on chlorovirus studies, is yet to be confirmed in prasinoviruses.

Previously, fibronectin‐binding protein was identified as a single copy gene in OlV1, OtV1 (Moreau et al., 2010) and OtV2 (Weynberg et al., 2011), but its role was not thoroughly investigated and was broadly categorised as “Miscellaneous.” Functional characterisation of this gene in MntV1 is difficult without gene knockout or knockdown experiments, leaving its specific role unclear. It remains to be determined whether this gene affects virion production, as suggested by its relation to the NFACT protein RNA binding domain, aids in attachment and entry via the fibronectin binding protein domain, or contributes to an entirely unknown cellular process.

Pfam domain analysis of the contracted orthogroups in MntV1, in comparison to the other nine prasinoviruses, identified 139 significant domains (Supplementary Table TS4C). Among these, a single gene family was reduced in number yet still retained in MntV1, which is the major capsid proteins (*mcp*). While most prasinoviruses have eight different copies of the *mcp* gene, MntV1 and *Bathycoccus* viruses each have only seven (Bachy et al., 2021; Moreau et al., 2010; Weynberg et al., 2011). The reason why certain prasinoviruses can assemble an icosahedral capsid with just seven *mcp*s, while others require eight to produce similarly shaped and sized capsids, remains unclear (Weynberg et al., 2017). The rest of the contracted gene families were completely absent in MntV1, as detailed in Supplementary Tables TS4C and TS4D. The genes absent in MntV1 include several genes involved in sugar transfer and metabolism (glycosyltransferase family 2, phosphofructokinase, NAD dependent epimerase/dehydratase, GDP‐mannose 4,6 dehydratase, thiamine pyrophosphate binding domain), genes carrying out nucleic acid and associated factor modifications (2OG‐Fe(II) oxygenase, AN1‐like Zinc finger domain, cytosine DNA methylase, Staphylococcal nuclease homologue) and others, involved in the production of dTTP (Cytidine and deoxycytidylate deaminase zinc‐binding region) and the biosynthesis of secondary metabolites (an aminotransferase), as well as several domains of unknown function. MntV1 likely employs different pathways or different genes to modulate the necessary pathways at different points compared to the other prasinoviruses, to achieve infection and propagation.

### Other gene differences

In order to gain a comprehensive understanding of MntV1, we performed functional annotation on all coding sequences within its genome and thoroughly examined the annotated Pfam domains. This approach provided us insights beyond those gained from Orthofinder by employing a genome‐wide annotation strategy. In this process, we identified 107 significant Pfam domain hits, with 29 (27.1%) domains being of unknown function.

To examine the gene conservation across prasinoviruses and chloroviruses, we compiled a comprehensive table (Supplementary Table TS5) detailing the gene copy numbers in 14 available fully annotated prasinovirus species and 6 chlorovirus species, incorporating findings from previous studies (Bachy et al., 2021; Finke et al., 2017; Moreau et al., 2010; Siotto et al., 2014; Weynberg et al., 2011), and the novel MntV1 functional annotations generated in this study. The genes vary widely, from those ubiquitous across all investigated viruses to those specific to either prasinoviruses or chloroviruses, as well as genes present in only a few taxa. We also incorporated annotations of tRNA genes obtained from the same studies and, for MntV1, from analysis with tRNAscan‐SE (Table TS5). MntV1, along with BpV1, has the lowest number of tRNA genes within the group tested, consisting only of the Asn‐tRNA and Ile‐tRNA found in all 14 prasinoviruses, and Leu‐tRNA, found in most.

Past investigations have identified 22 “core” genes considered universal among prasinoviruses and chloroviruses (Bachy et al., 2021; Derelle et al., 2015; Monier et al., 2017), 18 of which are pivotal for cellular functions and are commonly used for phylogenetic analyses. Alternatively, other research has highlighted other sets of “core” genes within the broader NCLDV family (Weynberg et al., 2009; Yutin et al., 2009), settling finally on 9 NCLDV “core” genes (Moniruzzaman et al., 2014, 2020). However, subsequent discoveries have revealed that some prasinovirus species lack a few genes from these “core” sets. As more viruses are studied, the diversity within the collective gene pool increases, leading to a smaller set of universally shared core. For our study, we merged several “core” gene sets (Derelle et al., 2015; Weynberg et al., 2009; Yutin et al., 2009) to define a revised consensus group of 23 genes that are prevalent in most prasinoviruses (Supplementary Table TS5). The newly characterised MntV1 contains all 23 genes from this curated list. Building upon this foundation, we propose the recognition of an additional seven genes that are also ubiquitous among prasinoviruses: RNAse H, exonuclease, glycosyltransferases, ATP‐dependent protease, FtsH metalloprotease, PhoH, and ABC‐1 domain protein. These genes comprise what we refer to as a “semi‐conserved” gene group, totalling 30 genes (listed in TS6). MntV1 possesses all of these “semi‐conserved” genes, confirming its genomic conservation within the prasinovirus lineage.

Manual gene comparison of MntV1 with other prasinoviruses and chloroviruses confirmed the presence of seven copies of the *mcp* gene in MntV1 and revealed unique genomic features that automated comparison methods had previously overlooked. First, MntV1 possesses a unique ubiquitin gene, which is present in a few chloroviruses (see Supplementary Table TS5; Moreau et al., 2010) and other NCLDVs (Iyer et al., 2006), but has not been observed in any other prasinoviruses. Ubiquitin is a small regulatory protein that plays a crucial role in eukaryotic cellular function by tagging proteins for degradation by the proteasome: a complex that disassembles unneeded or damaged proteins (Swatek & Komander, 2016). In eukaryotic viruses, ubiquitin and its associated pathways have been implicated in enhancing viral entry and replication (Schneider et al., 2021), a phenomenon observed in chloroviruses (Moreau et al., 2010; Noel et al., 2014). Genes involved in ubiquitination have been observed in all NCLDV clades (Moniruzzaman et al., 2014). Ubiquitin hydrolases, which detach ubiquitin from proteins, and ubiquitin ligases, which attach ubiquitin to target proteins (Derelle et al., 2018), have been found in several prasinoviruses (Moreau et al., 2010), which suggests that prasinoviruses leverage the host ubiquitin‐proteasomal system in multiple ways. MntV1's expression of its own ubiquitin protein could represent a novel strategy within prasinoviruses. Further investigation is required to unravel the precise function and significance of the ubiquitin gene in prasinoviruses.

Glycerophosphoryl diesterase (GPD), another gene unique to MntV1 among prasinoviruses, is also found in some chloroviruses (Moreau et al., 2010) and other NCLDVs (Blanc‐Mathieu et al., 2021). This enzyme is crucial in chlorovirus phospholipid metabolism, hydrolysing glycerophosphodiesters, which are key components of host cell membranes. This breakdown releases glycerol and phosphate groups, which are then leveraged by the virus for replication, assembly, and egress (Cornelissen et al., 2016). Additionally, GPD may modulate host cell metabolism by altering the lipid composition of the cell membranes, potentially impairing host defence responses and creating an optimal environment for viral proliferation (Hejazian et al., 2024). The presence of a GPD gene in MntV1 highlights its potential significance in viral life cycle and host interaction dynamics.

### Gene transfer analysis

To examine the evolutionary relationship between MntV1 and its algal host, *Mantoniella tinhauana*, we performed a blastp analysis. This bioinformatic approach is designed to uncover possible genetic exchanges that may have occurred between the virus and its host. The analysis aimed to detect viral proteins with considerable sequence alignment to host‐encoded proteins, applying stringent criteria: ≥ 50% amino acid identity and ≤1 × 10^−15^ E‐value (Table TS7). Our analysis yielded four viral genes that fit these criteria, suggesting a selective and potentially ancient gene transfer event. The modest number of similar genes and the relatively low percentage of identity suggest that these putative gene transfer events are not recent, and that the viral genes have likely diverged extensively over time, possibly due to mutation or recombination. Moreover, when extending the comparison of these four viral genes against the broader NCBI‐nr database, which includes a vast array of sequences from various organisms, we found additional significant matches. Notably, the ubiquitin gene stood out as the only one exhibiting both a higher match identity and a more significant E‐value when comparing virus–host with virus‐NCBI‐nr database hits (Supplementary Table TS8). This suggests a closer evolutionary relationship (and more recent gene exchange) between the MntV1 virus and its host for this gene compared to the rest of its genes.

**FIGURE 4:**
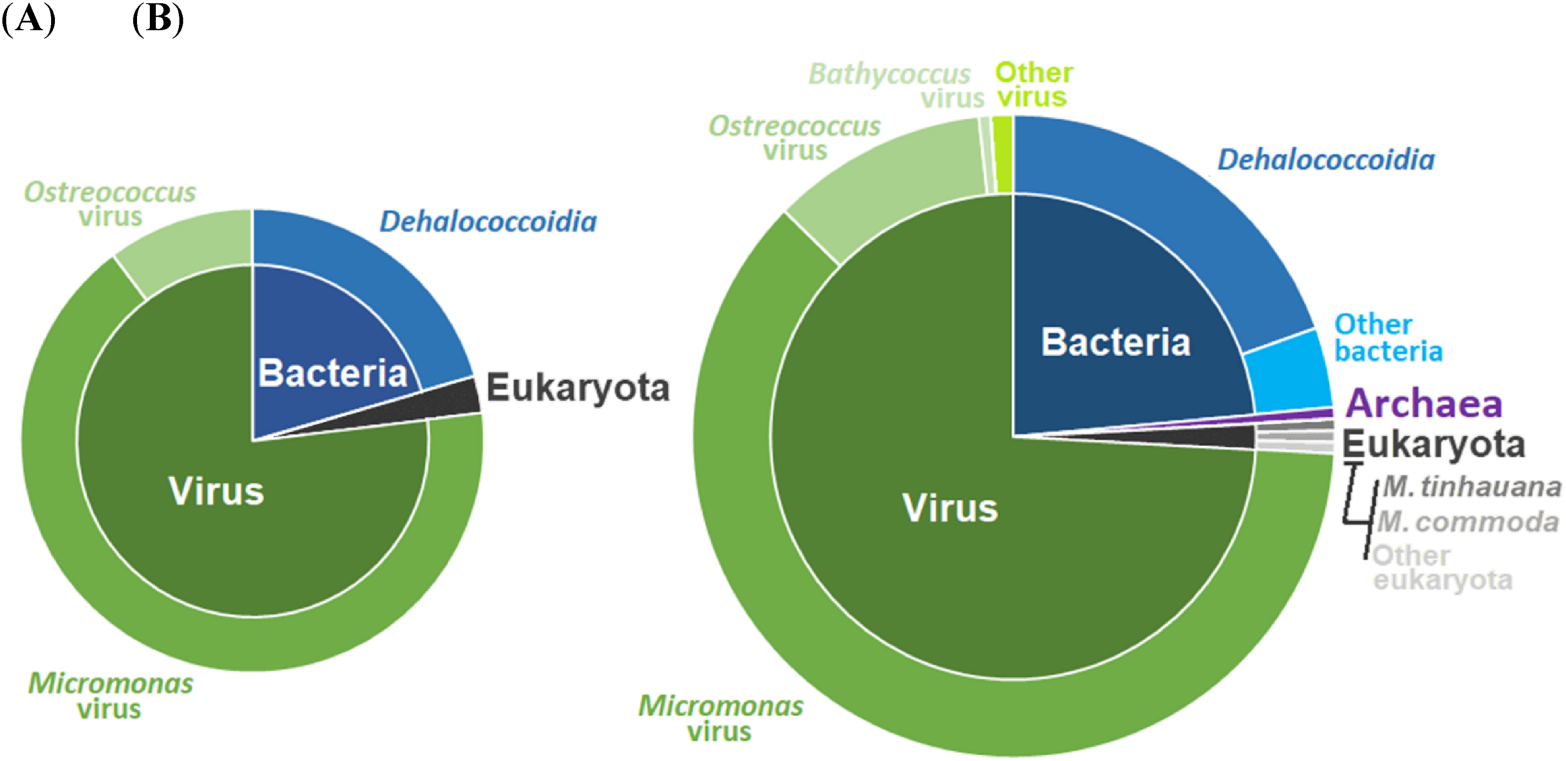
Presumed source organism of MntV1 genes based on blastp best hits against the NCBI‐nr database and *M. tinhauana*. (A) Source of MntV1's “semi‐conserved” prasinovirus genes. (B) Source of all MntV1 genes with blastp hits.

To explore the possibility of gene acquisitions from other sources like bacteria or alternative hosts, we extended our blastp search to include the comprehensive NCBI‐nr database using the same ≥50% amino acid identity and ≤1 × 10^−15^ E‐value thresholds. This broader search yielded 175 high‐confidence matches, which we scrutinised for source organism identification and functional annotation (Supplementary Table TS8). Many matches were to bacterial genes, with 34 hits corresponding to genes from *Dehalococcoidia*, a bacterial class prevalent in marine subsurface sediments (Wasmund et al., 2014). Additionally, one match aligned with a *Euryarchaeot*a archaeon, three with eukaryotic species (including two from Mamiellophyceae class: *M. tinhauana* and *Micromonas commoda*), two with non‐prasinovirus viruses, and 126 with other prasinovirus sequences. Among the prasinovirus‐related hits, MpV1 emerged as the primary match, with 89 genes. The distribution pattern of the source organisms for the “semi‐conserved” prasinovirus genes subset (Figure 4A and Table TS6) was very similar to that of the entire genome (Figure 4B and Table TS8).

Previous studies also determined that prasinoviruses and other NCLDVs have a very significant percentage of genes of bacterial origin, obtained via horizontal gene transfer (HGT) (Filée, 2015; Finke et al., 2017; Moniruzzaman et al., 2014). Our results show a higher proportion of viral matches compared to earlier studies, alongside fewer hits to prokaryotic and eukaryotic origin matches. This shift is likely due to the recent expansion of viral sequences in reference databases. The data suggest a history of extensive ancient HGT from bacteria and some eukaryotes to prasinoviruses, with their descendants frequently undergoing recombination (Clerissi et al., 2013; Yau, Krasovec, et al., 2020). Additionally, more isolated and recent HGT events appear in specific prasinovirus clades. These are accompanied by gene losses, as illustrated in the expansions and contractions analysis (Figure 3), which help maintain a small genome size. In contrast, other NCLDVs such as mimiviruses and chloroviruses display different evolutionary patterns, characterised by fewer HGT events—though there are notable exceptions (Monier et al., 2009)—and more frequent duplications, deletions, and rearrangements (Filée, 2015).

**FIGURE 5:**
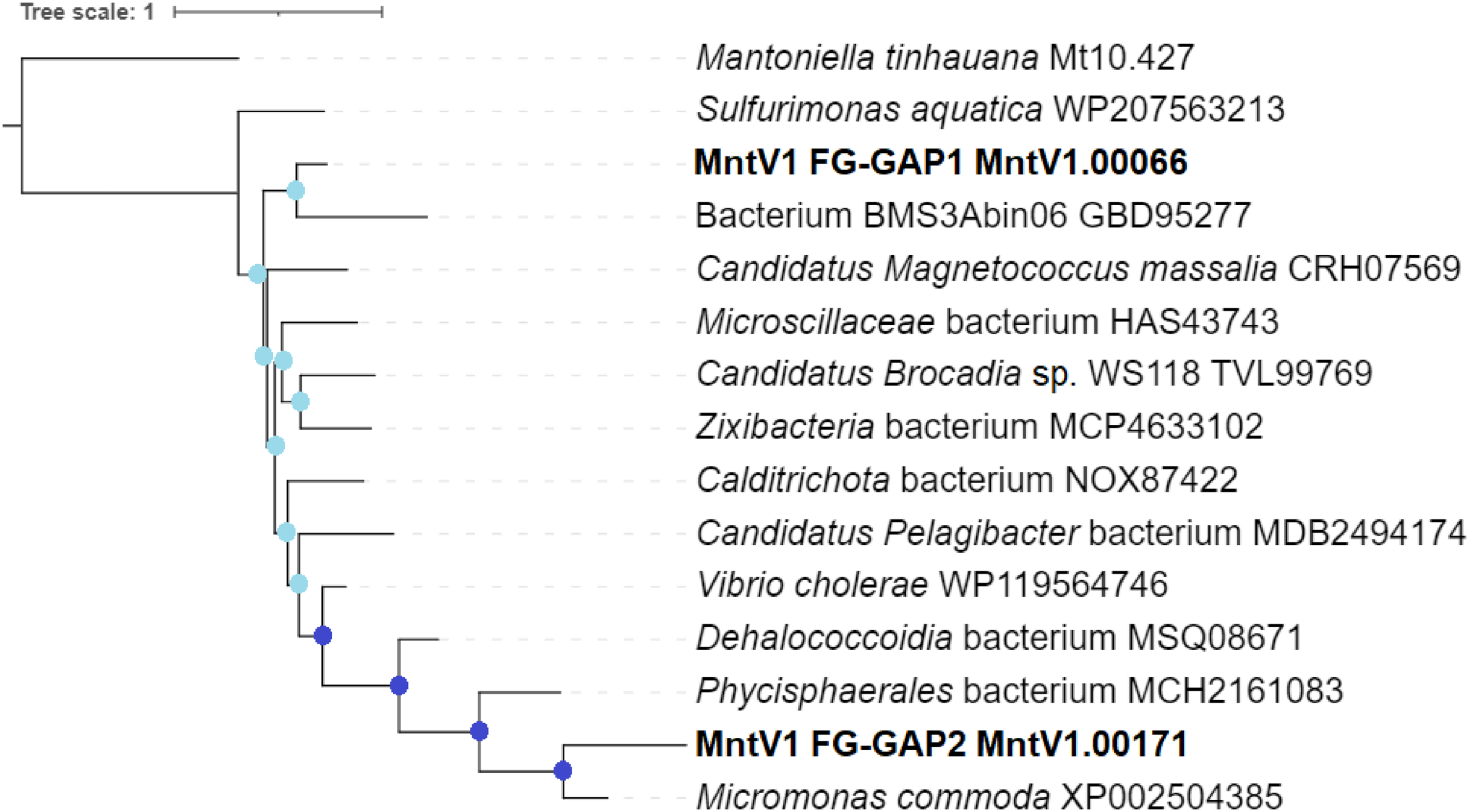
ML tree of best MntV1 FG‐GAP gene blastp hits, with the two MntV1 genes in bold. Nodes with sufficient support (>80% Shimodaira‐Hasegawa [SH]‐like approximate likelihood ratio test [aLRT] and >95% Bayesian posterior probability) are marked in dark blue, whereas those without sufficient support are in light blue. Gene ID/accession numbers are included.

The two MntV1‐unique genes identified by Orthofinder, which are the FG‐GAP repeat domain sequences discussed above, had matches to a host gene located on chromosome 10 albeit below our defined similarity threshold. It is reasonable to assume that a gene facilitating specific integration into a host cell could have been acquired through gene transfer from that host cell. The two viral copies showed only 29% similarity to each other. One had its best match to *Micromonas commoda* FG‐GAP and the other to a bacterial FG‐GAP gene, with significantly higher identity than the host gene, as seen in Figure 5. One possible explanation for this is that the viral host initially acquired the FG‐GAP repeat gene from a bacterial source, which was then transferred to MntV1. Subsequent divergent selective pressures on the host and virus may have resulted in the viral gene retaining more similarity to the original bacterial gene. The observation that *M. commoda* aligned better than *M. tinhauana* with the viral gene indicates that MntV1 may have once been capable of infecting *M. commoda* and acquired a copy of FG‐GAP at that time, an ability that our host range experiments suggest has since been lost. It is worth noting that the FG‐GAP domain gene of medusavirus, the only other viral example identified, did not match any MntV1 sequences in our blastp search (Yoshikawa et al., 2019). A manual alignment of the medusavirus FG‐GAP with the two FG‐GAP sequences from MntV1 showed almost no identity, raising questions about potential misannotation of their ORF. When conducting a blastp search for the medusavirus FG‐GAP, the results included a range of proteins with surface receptor domains, and several matches were more significant than those for bacterial FG‐GAPs, which showed low identity at 26%. This suggests that MntV1 might actually be the first virus confirmed to possess FG‐GAP repeat domains.

Our analysis of the expanded NFACT protein RNA‐binding domains in MntV1 revealed no significant homology with the host genome, yielding only low‐quality hits. Intriguingly, one of the NFACT protein copies had a significant hit to a *Betaproteobacteria* protein containing the DUF814 domain, while the other copy aligned with an OlV4 hypothetical protein, although with less than 50% identity. Proteins with NFACT RNA‐binding, DUF814, and fibronectin‐binding domains are functionally linked and have been found in all domains of life (Burroughs & Aravind, 2014). This suggests that MntV1, along with other prasinoviruses, likely acquired these domains from a bacterial origin, subsequently undergoing divergent evolution in different lineages. The MntV1‐unique GPD gene discussed above had no significant blastp hits, so no inferences could be made on its origin.

Upon comparison of MntV1's genomic sequences against the NCBI‐nr database, only four significant eukaryotic matches were identified. This paucity of eukaryotic sequences might reflect a bias within the database towards prokaryotic sequences, a limitation commonly highlighted in the literature (del Campo et al., 2014). The underrepresentation of eukaryotic genetic material, particularly that of unicellular eukaryotes, could imply that gene transfers from eukaryotic sources to MntV1 might be more prevalent than our results indicate. Nevertheless, due to the scarcity of comprehensive eukaryotic genomic annotations, bacterial sequences—which are typically better characterised—tend to dominate as the closest matches in our data. It is also possible that MntV1 has experienced extensive gene transfer from bacterial sources. Despite the considerable volume of viral data available, the lack of detailed functional annotations often means that viral sequences with unknown roles could conceal other more functionally informative sequence matches. Hence, the current analysis may not fully capture the extent of eukaryotic gene transfer events that have shaped the MntV1 genome, underscoring the need for enhanced eukaryotic sequence representation and annotation in genomic databases.

### Global biogeography

**FIGURE 6:**
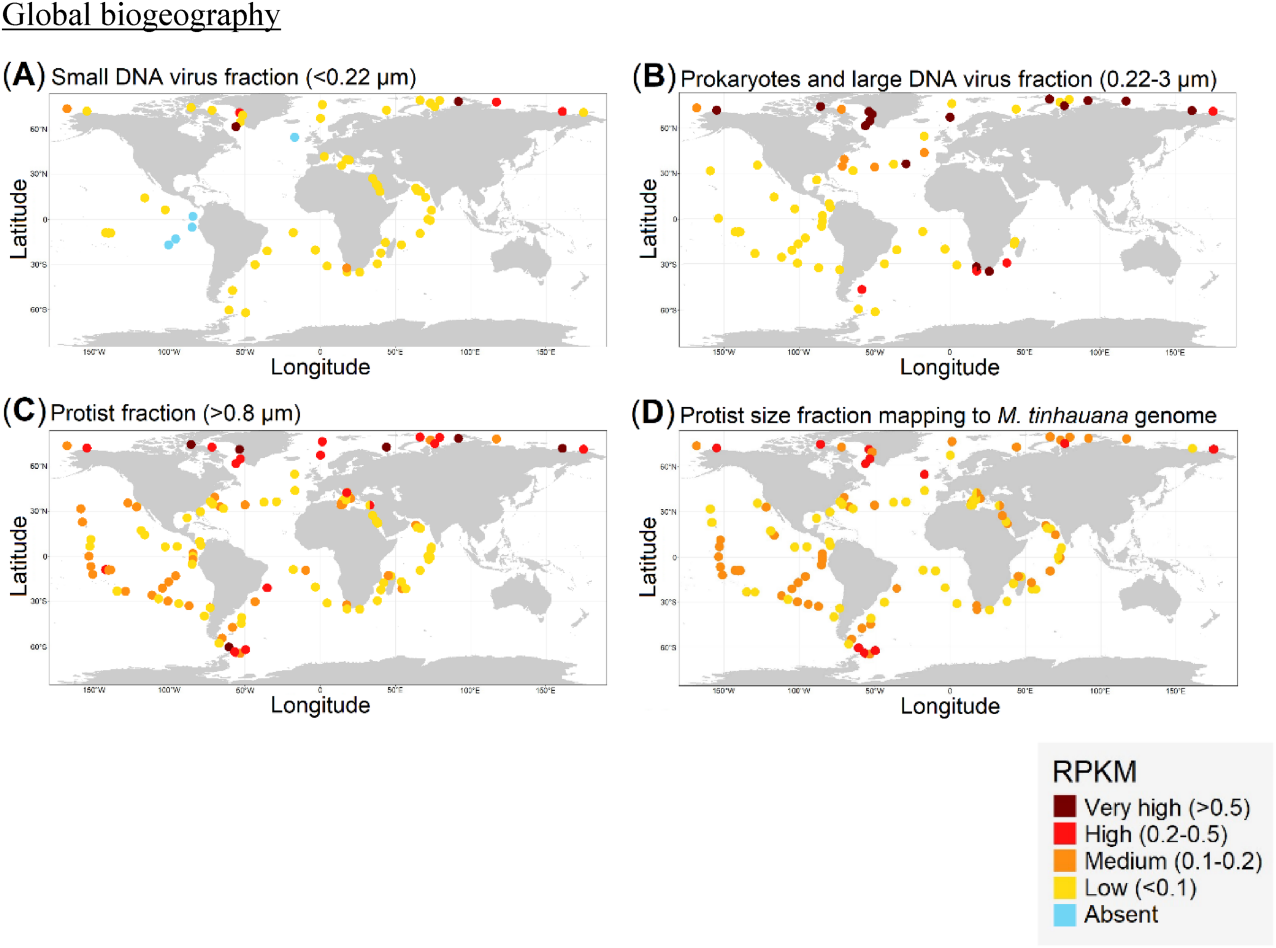
Maps of different size fraction global metagenomic read mapping to viral and host reference genomes. (A) Small DNA virus (<0.22 μm) fraction read mapping to MntV1. (B) Prokaryote and large DNA virus (0.22–3 μm) fraction read mapping to MntV1. (C) Protist (>0.8 μm) fraction read mapping to MntV1. (D) Protist fraction read mapping to *M. tinhauana*.

Analysis of global metagenomic samples fractionated by particle size provided insights into the distribution and ecological dynamics of MntV1. We examined three size fractions for the relative abundance of MntV1 genomic reads at minimum 95% identity and found MntV1 in nearly all sampled locations (Figure 6). While not every location was covered in each size fraction, most were represented across all three.

The smallest size fraction (<0.22 μm, Figure 6A) corresponds to the size range of individual MntV1 virions (averaging 0.12 μm in diameter as determined by TEM) and mostly excludes bacteria and eukaryotic cells. Within this fraction, MntV1 was detected at the lowest relative abundance among the size fractions (median RPKM across all sites = 0.004, mean = 0.099, Supplementary Table TS9) and completely absent from some locations. This suggests that a considerable proportion of MntV1 may exceed the size threshold due to viral aggregation or that these virions may predominantly exist within their host cells rather than as free particles. The variability within this fraction was highlighted by a high standard deviation (Supplementary Table TS9). Remarkably, the sites in the Kara Sea (2.970 RPKM, highest among all sites and fractions) and Labrador Sea (1.096 RPKM) showed exceptionally high RPKM values. The disproportionate abundance of free MntV1 particles in these sites might reflect an intrinsically high viral abundance, encompassing both free virions and those within host cells. Alternatively, the sample collection may have coincided with an event of viral lysis, resulting in the release of a high number of virions into the environment.

The intermediate size fraction (0.22–3 μm, Figure 6B), capturing prokaryotes and large DNA viruses, had a higher mean MntV1 RPKM (mean RPKM = 0.249) with the highest standard deviation among the fractions (SD = 0.422, Supplementary Table TS9). All sites tested contained MntV1 within this fraction. Given that *M. tinhauana* cells range from 1.8 to 4.5 μm in diameter (median 3.1 μm) (Rey Redondo et al., 2024), this fraction likely includes half of the host cells present in the water samples. The cell size distribution of *M. tinhauana* across different environments remains unclear, which introduces a degree of uncertainty in using this fraction to reliably indicate the presence of MntV1 or its host. The range of RPKM values in this fraction was characterised by considerable heterogeneity, skewing towards either notably low or exceedingly high values. This pronounced polarisation in RPKM distribution was especially evident in coastal and polar sites. For example, the Kara Sea exhibited a peak RPKM of 2.649 for MntV1, aligning with the previously noted high abundance of MntV1 in its free form. This suggests that in such regions, there may be an increased density of *M. tinhauana* cells, potentially leading to more frequent and dynamic host‐virus interactions. The observed variability, particularly the high RPKM values, might indicate localised blooms of the host cells, possibly triggered by environmental conditions conducive to their proliferation, which in turn could facilitate a rise in viral activity and abundance.

The largest size fraction (>0.8 μm, Figure 6C) includes all measured *M. tinhauana* cell sizes and is less likely to contain non‐cell‐associated MntV1 virions. MntV1 was consistently detected across all environments in this fraction, with a higher median RPKM (0.104) and a more moderate maximum value (1.268 RPKM) compared to smaller fractions (Supplementary Table TS9). This consistent detection indicates that this fraction is a reliable marker of active infections within *M. tinhauana* populations, as opposed to the other fractions that may contain both free virions and intracellular ones. Notably, while MntV1 is generally widespread at a low abundance, its prevalence is markedly higher in the polar regions, indicating a possible relationship with the ecological or climatic conditions that influence viral‐host dynamics in these extreme environments.

In an effort to elucidate the global distribution and interaction of MntV1 and its host *M. tinhauana*, we conducted an analysis of metagenomic sequences from the protist size fraction (>0.8 μm), targeting the host's genomic signatures (Figure 6D). The observed distribution pattern for *M. tinhauana* was strikingly akin to that of the MntV1 RPKM values obtained from the same protist metagenomic dataset. However, the host genome data exhibited a more constrained range of values, with a reduced incidence of outliers, resulting in a lower standard deviation across the sampled sites. The median value of host genome mappings within the protist size fraction was found to be almost identical to the median value of the viral mappings (Supplementary Table TS9). The relationship between the abundance of host and virus within this fraction was quantified using SRCC, showing a strong positive correlation (SRCC = 0.71, *p*‐value = 0, Supplementary Figure S3A). This significant correlation was anticipated, as it is likely that the majority of viral reads originate from MntV1 particles that are within or attached to their host cells.

To elucidate the interplay between the distribution of MntV1 and its host *M. tinhauana*, and environmental parameters, we scrutinised the environmental variables in conjunction with the viral and host RPKM data. Owing to the high degree of collinearity (>50%) among most variables, we reduced the environmental dataset to three key variables with minimal collinearity: temperature, salinity, and nitrate concentration. The SRCCs for these variables were similar for both virus and host RPKM (Supplementary Figure S3B). In our analysis, temperature emerged as the environmental factor with the strongest negative correlation with the distributions of both virus and host, followed by salinity, which also demonstrated a negative correlation. Conversely, nitrate concentration was positively correlated with the presence of MntV1 and *M. tinhauana*. The significant virus RPKM‐environmental factor correlations for the other two size fractions are summarised in Supplementary Figure S3C,D. Notably, the highest correlation was observed between MntV1 RPKM and temperature (−0.49 SRCC). This finding aligns with the observed trend of higher MntV1 abundances in cooler polar waters and lower concentrations in the warmer equatorial regions (Figure 6C). Furthermore, the high‐latitude areas, typically characterised by higher nitrate levels and lower salinity, matched the observed distribution patterns of both MntV1 and *M. tinhauana* (Figure 6C,D), suggesting a multifactorial environmental influence on their distributions.

## CONCLUSIONS

We report the isolation and characterisation of *Mantoniella tinhauana* virus 1 (MntV1), a novel prasinovirus from the coastal waters of Hong Kong, which infects the green alga *Mantoniella tinhauana*. TEM revealed icosahedral virions typical of prasinoviruses, with observed aggregations and adherence to host scales, as of yet of undetermined consequence. Phylogenetic analysis using *polB* and NCLDV marker genes places MntV1 firmly within the prasinovirus clade, closely related to viruses infecting *Micromonas* spp. and *Ostreococcus* spp. Furthermore, cross‐infection experiments confirm that MntV1 exhibits a highly specific host range, with only one host species identified to date.

The MntV1 genome conforms to the typical characteristics of prasinovirus genomes in terms of size, GC content, and coding sequence count, albeit on the smaller end of the spectrum. No TIRs were identified, which sets MntV1 apart from other prasinoviruses. Comparative genomic analysis revealed that MntV1 shares many orthogroups with other prasinoviruses but has fewer overall, suggesting modest genomic streamlining. Despite having a reduced set of tRNAs, MntV1 retains three tRNAs found in most prasinoviruses, and the 30 “semi‐conserved” prasinovirus proteins, confirming its identity within this group while also exhibiting unique genomic features.

The MntV1 genome features three genes absent in other known prasinoviruses, indicating unique evolutionary adaptations. The FG‐GAP repeat‐containing gene, which shares homology with bacterial and *Micromonas commoda* genes, might represent a novel viral attachment or entry mechanism, thus warranting further investigation (Al‐Shami et al., 2013; Sun et al., 2014). The presence of the ubiquitin gene in MntV1, also found in some chloroviruses and other NCLDVs (Iyer et al., 2006; Moreau et al., 2010), likely results from HGT from *Mantoniella tinhauana*. This suggests that MntV1 may have developed a unique mechanism within prasinoviruses to modulate the host's ubiquitin‐proteasome system, potentially enhancing viral replication or evading host defences (Schneider et al., 2021; Swatek & Komander, 2016). Additionally, the presence of a gene encoding GPD (also present in some chloroviruses and other NCLDVs [Blanc‐Mathieu et al., 2021; Moreau et al., 2010]), associated with cell surface attachment, further reinforces the notion of an alternative infection strategy for virus entry into host cells employed by MntV1 among the prasinoviruses. Detailed functional analysis of these genes is essential to unveil their specific roles in virus–host interaction.

Comparative gene family evolution analysis reveals that MntV1 has undergone notable genomic reductions with a few expansions in comparison to other prasinoviruses. Specifically, MntV1 possesses an additional copy of an NFACT/fibronectin‐binding protein gene, which could represent an evolutionary adaptation of potential interest. Contrastingly, MntV1 displays a reduction in the number of major capsid protein genes, with only seven compared to the more common eight found in other prasinoviruses (Moreau et al., 2010). This discrepancy, however, does not affect the overall capsid structure, which is conserved within the group. The reduced *mcp* gene count may be linked to the unique scale‐covered morphology observed in *Mantoniella* and *Bathycoccus* (Moreau et al., 2012; Yau, dos Santos, et al., 2020), the only scaled genera among Mamiellophyceae algae. Further research into how these capsid protein differences interact with the scaled surface of the algae may elucidate specific viral mechanisms for glycan‐mediated attachment and entry.

The gene composition of MntV1 largely mirrors that of other prasinoviruses, indicating a strong shared lineage within this group. The high proportion of genes related to bacterial sequences, typical of many NCLDVs, might suggest past HGT events from bacteria to the virus. Conversely, the sparse eukaryotic sequence matches may reflect either few HGT events between eukaryotes and MntV1 or an underrepresentation of eukaryotic genomes in existing databases (del Campo et al., 2014). To further elucidate the evolutionary dynamics between MntV1 and marine algal hosts, it is crucial to expand genomic databases with more comprehensive data, especially from the Mamiellophyceae, including known *Mantoniella* species as well as other as yet unsequenced or uncultivated strains, and more prasinovirus genomes MntV1 might have recombined with (Clerissi et al., 2013; Yau, Krasovec, et al., 2020). Such enhancements will improve the detection of HGT events and provide greater insight into the co‐evolution of marine viruses and their hosts, as well as the broader ecological and evolutionary processes influencing marine microbial communities.

The lack of a small outlier chromosome (SOC) in *Mantoniella tinhauana* complicates the identification of virus resistance genes, since SOCs in other species often contain adaptive genetic traits for pathogen resistance (Moreau et al., 2012). This absence hinders direct comparisons with other Mamiellophyceae species, like *Ostreococcus*, whose SOCs show genomic adaptations for viral defence such as codon rearrangements and gene losses (Yau et al., 2016; Yau, Krasovec, et al., 2020). The absence of an SOC in *Mantoniella tinhauana* suggests that this species might utilise widespread, nuanced genomic modifications or a broader, non‐specific immune response, potentially relying on non‐genomic defences like RNA interference or other post‐transcriptional regulatory mechanisms (Ding, 2010). Consequently, creating a MntV1‐resistant *M. tinhauana* strain to study potential resistance mechanisms, if possible, would provide much insight (Thomas et al., 2011; Yau et al., 2016; Yau, Krasovec, et al., 2020). Such research could include analysing gene expression changes in MntV1‐infected versus uninfected cultures to better understand virus–host interaction dynamics.

A metagenomic investigation into the global distribution patterns of MntV1 and its host, *Mantoniella tinhauana*, revealed a worldwide distribution with significant regional variations in abundance. Notably, both the virus and the alga exhibit a marked co‐occurrence, reaching maximal densities within the polar regions, characterised by colder temperatures, reduced salinity levels, and elevated nitrate concentrations. These conditions appear to have driven a co‐evolutionary adaptation process in MntV1 and *Mantoniella tinhauana*, influencing their metabolic activities and interaction dynamics. The unique ecological niches created by the influx of cold freshwater from ice melts and riverine systems, and nutrients from upwelling or runoff are particularly conducive to the proliferation of *M. tinhauana*. This proliferation, in turn, appears to dictate the distribution of MntV1, illustrating a dependency of the virus on the algal host's life cycle and abundance. This close relationship between MntV1 and *M. tinhauana* in such environments underscores a delicate equilibrium, where the virus–host dynamic is intimately tied to the specific habitat conditions. This relationship offers profound insights into the role of abiotic factors in shaping microbial and viral distribution and emphasises the interconnectedness of viral and host survival and adaptation strategies.

## AUTHOR CONTRIBUTIONS

**Elvira Rey Redondo:** Conceptualization; methodology; investigation; validation; formal analysis; visualization; writing – review and editing; writing – original draft. **Shara Ka Kiu Leung:** Investigation; methodology. **Charmaine Cheuk Man Yung:** Conceptualization; methodology; supervision; resources; project administration; writing – review and editing; writing – original draft; funding acquisition; investigation; validation.

## CONFLICT OF INTEREST STATEMENT

The authors declare that there is no conflict of interest.

## Supporting information


**FIGURE S1.** Culture_Lysis.
**FIGURE S2.** polB_MLtree.
**FIGURE S3.** SRCC.

**TABLE S1.** Host_range.
**TABLE TS2**. Genomes.
**TABLE TS3**. Orthofinder.
**TABLE TS4**. Pfam.
**TABLE TS5**. Gene_copies.
**TABLE TS6**. Semi‐conserved.
**TABLE TS7**. Host_blast.
**TABLE TS8**. Gene_transfers.
**TABLE TS9**. RPKM_dist.

## Data Availability

The annotated MntV1 genome was deposited to GenBank under accession PP130629.
