# Supplementary material for: Genomic and biogeographic characterisation of the novel prasinovirus Mantoniella tinhauana virus 1
Source: Environ Microbiol Rep. 2024 Oct 11;16(5):e70020. doi: 10.1111/1758-2229.70020 (PMC11467894; doi:10.1111/1758-2229.70020)
Supplement: Supplementary file 1 — FIGURE S1. Culture_Lysis. FIGURE S2. polB_MLtree. FIGURE S3. SRCC. [file EMI4-16-e70020-s001.docx]

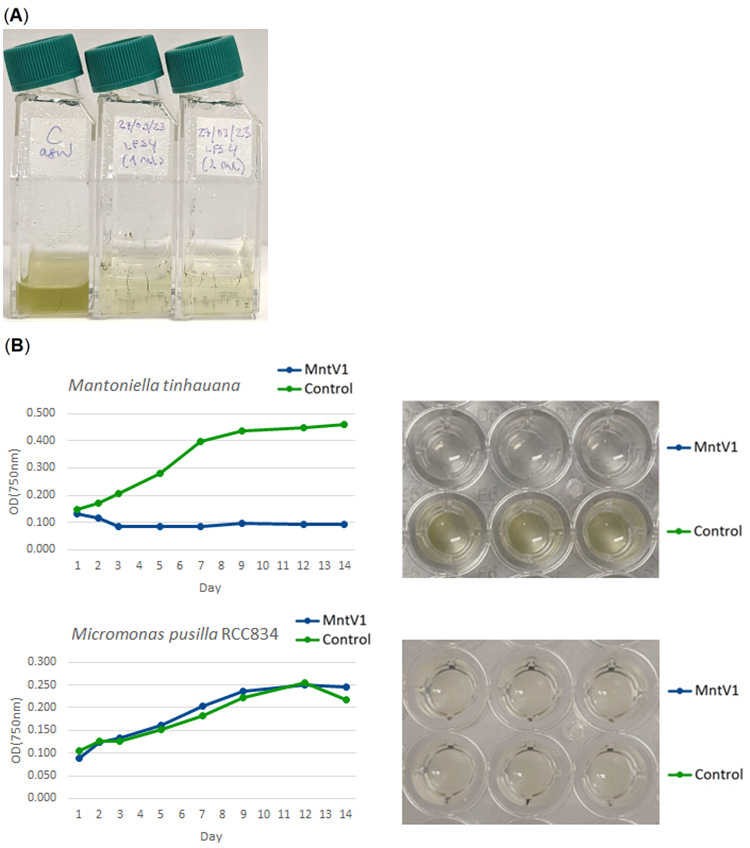


**Supplementary Figure S1.** Visible lysis of algal culture by MntV1. (**A**) Photograph of three *Mantoniella tinhauana* cultures, the first with 1 ml artificial seawater added to the 10 ml culture four days prior as a control, the second with 1 ml of MntV1 lysate added at the same time, and the third with 2 ml added. MntV1 infection visibly lyses the *M. tinhauana* cells, making the culture go from green to transparent. (B) Sample results of the (host range) microplate lysis experiments. Graph of (triplicate means) optical density measurements at 750 nm over two weeks, with MntV1 addition versus artificial seawater addition, and photos of the microplate cultures at day 14 (the last day) for visual representation of lysis. The graph and microplate for *M. pusilla* are included for comparison purposes (no MntV1 lysis).


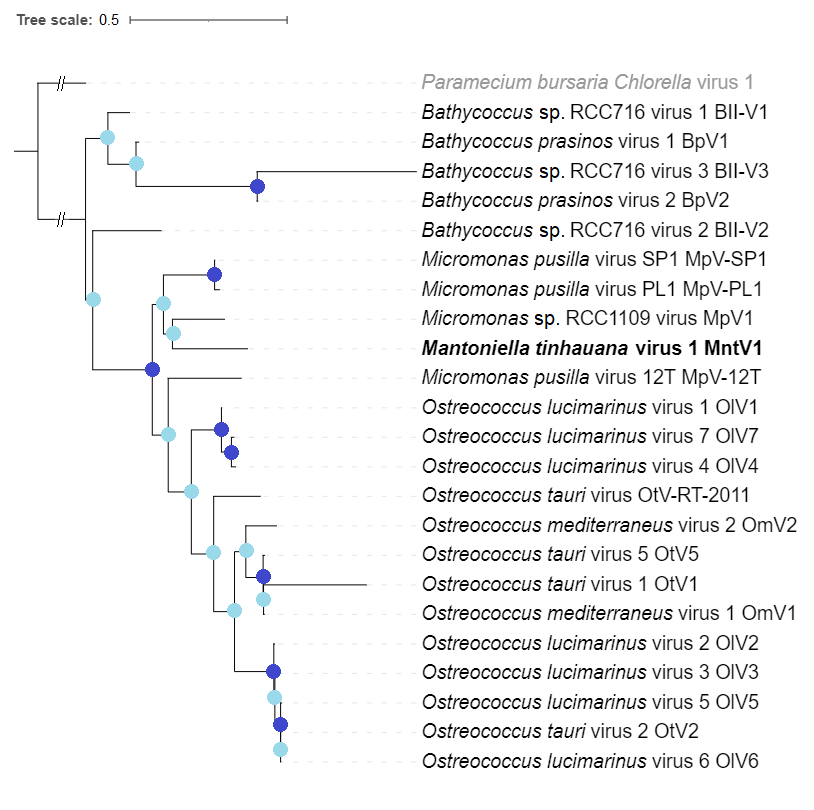


**Supplementary Figure S2.** Maximum Likelihood prasinovirus partial *polB* tree. *Paramecium bursaria Chlorella* virus 1 was used as an outgroup (grey), and the novel MntV1 is highlighted in bold. Nodes with sufficient support (>80% SH-like aLRT and >95% Bayesian posterior probability) are marked in dark blue whereas those without sufficient support are in light blue.


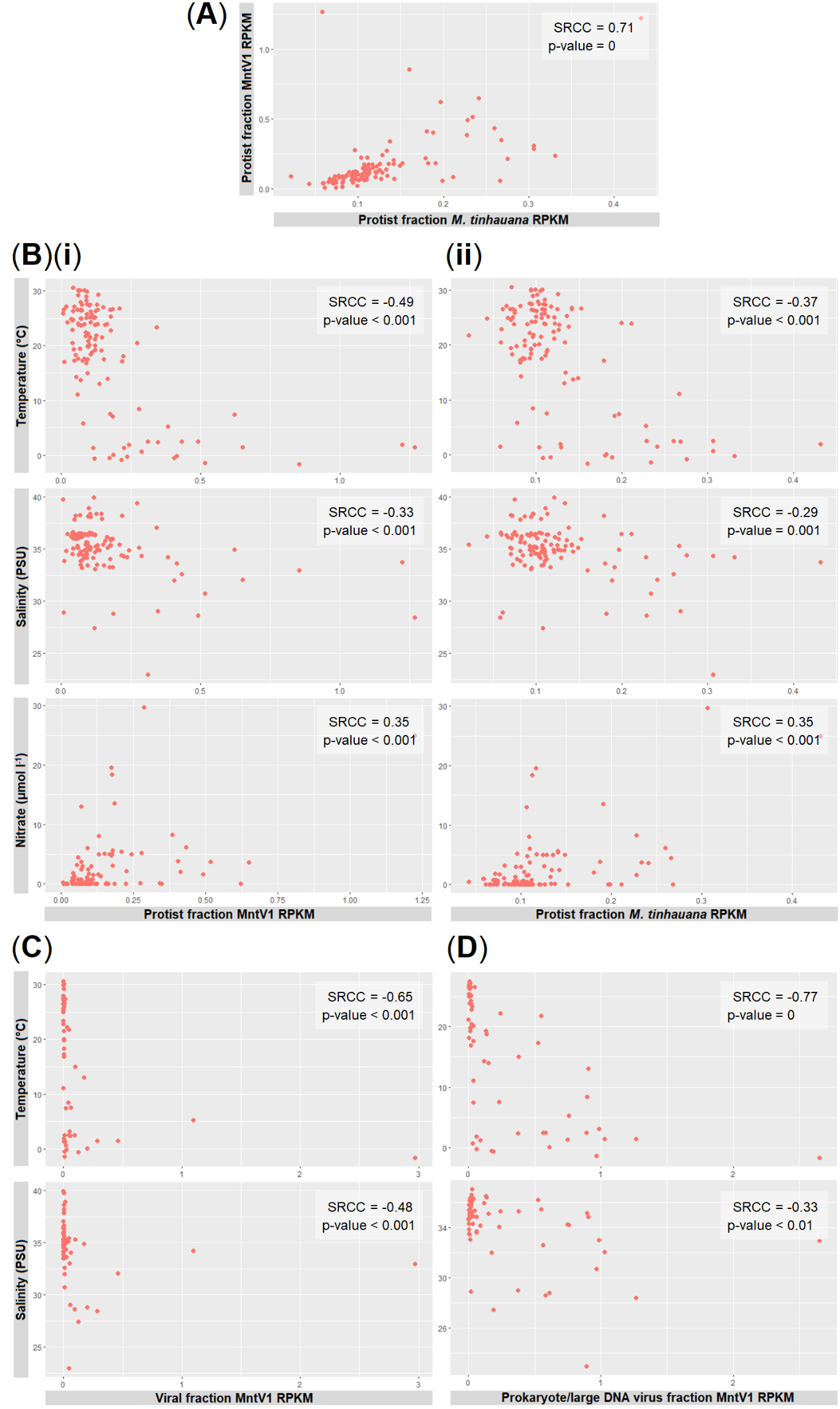


**Supplementary Figure S3**. Scatterplots of virus-host and significant virus/host-environmental factor correlations. SRCC values and their corresponding p-values are shown. (**A**) Correlation of *M. tinhauana* RPKM with MntV1 RPKM in the protist fraction read mappings. (**B**) Protist fraction environmental factor-RPKM correlations, (**i**) correlation of MntV1 RPKM with environmental variables, (**ii**) correlation of *M. tinhauana* RPKM with environmental variables. (**C**) Viral fraction significant correlations between environmental variables and MntV1 RPKMs. (**D**) Prokaryote/large DNA virus fraction significant correlations between environmental variables and MntV1 RPKMs.
